# Analyzing the functional effects of DNA variants with gene editing

**DOI:** 10.1016/j.crmeth.2024.100776

**Published:** 2024-05-13

**Authors:** Sarah Cooper, Sofia Obolenski, Andrew J. Waters, Andrew R. Bassett, Matthew A. Coelho

**Affiliations:** 1 Cellular and Gene Editing Research, Wellcome Sanger Institute, Hinxton, UK; 2 Experimental Cancer Genetics, Wellcome Sanger Institute, Hinxton, UK; 3 Department of Dermatology, Leiden University Medical Center, Leiden, the Netherlands; 4 Cancer Genome Editing, Wellcome Sanger Institute, Hinxton, UK

## Abstract

Continual advancements in genomics have led to an ever-widening disparity between the rate of discovery of genetic variants and our current understanding of their functions and potential roles in disease. Systematic methods for phenotyping DNA variants are required to effectively translate genomics data into improved outcomes for patients with genetic diseases. To make the biggest impact, these approaches must be scalable and accurate, faithfully reflect disease biology, and define complex disease mechanisms. We compare current methods to analyze the function of variants in their endogenous DNA context using genome editing strategies, such as saturation genome editing, base editing and prime editing. We discuss how these technologies can be linked to high-content readouts to gain deep mechanistic insights into variant effects. Finally, we highlight key challenges that need to be addressed to bridge the genotype to phenotype gap, and ultimately improve the diagnosis and treatment of genetic diseases.

## Teaser

The functional consequences and disease relevance of most DNA variants found in human genomes are unknown. Here, Cooper et al. review CRISPR-based gene editing approaches that enable scalable phenotyping of disease variants by directly installing DNA variants into the genome, such as saturation genome editing, base editing, and prime editing.

## Introduction

The research community is sequencing human genomes at an accelerating rate, and these genomic data have exciting potential to identify links between genetic variation and human diseases. These efforts have focused on investigating genetic variation across hundreds of thousands of healthy individuals and smaller cohorts of patients with genetic diseases. Recently, the UK Biobank released almost 500,000 whole-genome sequences.^1^ Additionally, the Genome Aggregation Database (gnomAD)^2^^,^^3^ now comprises 76,156 human genomes, identifying over 16 million missense variants from genome and exome databases combined. For genetic diseases such as cancer, international consortia have collectively sequenced 2,658 genomes from cancer samples.^4^ Collectively, these resources have been transformative in discovering rare variants, informing genome-wide association studies (GWASs), and understanding the mechanistic basis of genetic diseases. However, the burden of missense variants in disease-relevant genes considered as variants of unknown significance (VUS) currently stands at 50%–98%.^5^^,^^6^^,^^7^ Furthermore, there are currently more than 560,000 variant to phenotype associations from GWASs,^8^ which are mostly from non-coding disease-related single-nucleotide variants (SNVs), expression quantitative trait loci (eQTLs), and require functional validation to prove causality. This highlights the mounting challenge of understanding DNA variant function at scale, making causative links from correlative genomic studies. An improved understanding of variant effects could lead to better genetic diagnosis and a deeper understanding of disease biology, thus informing the development of better drugs and patient stratification for therapy. A retrospective analysis of AstraZeneca’s drug discovery pipeline showed that, when human genetic data were available to link drug targets to disease, drug development campaigns were less likely to fail; 73% of active phase II trials had strong genetic evidence, compared to 43% of failed projects.^9^

Assessing DNA variant effects in their endogenous DNA context is important for several reasons. For protein-coding variants, overexpression from cDNA libraries could have profound and potentially confounding effects on the assayed variant function, especially if this disrupts native protein-protein interactions or the stoichiometry of protein complexes.^10^^,^^11^^,^^12^^,^^13^ Furthermore, the effect of splice variants can be missed, and more complex variants in gene-regulatory elements are difficult to analyze without prior in-depth knowledge of the functional architecture of the surrounding chromatin. Lastly, for investigating variants in the 98% of the human genome that is non-coding, endogenous genomic context is critical to faithfully measure potential changes in gene expression and function without compromising the chromatin landscape and poorly understood long-range genetic interactions.

The functional analysis of SNVs was once carried out on a one-by-one basis and could take years of a research scientist’s time. Presently, experiments analyzing variant function can be performed rapidly, in a pooled manner, and at a much larger scale, partly owing to significant technological advancements and cost reductions in next-generation sequencing (NGS) and DNA synthesis for the generation of variant libraries. Importantly, these multiplexed assays of variant effects (MAVEs)^14^^,^^15^ allow for internal benchmarking to known benign and pathogenic controls and for direct comparisons between query variant effects.^16^^,^^17^

**Figure 1:**
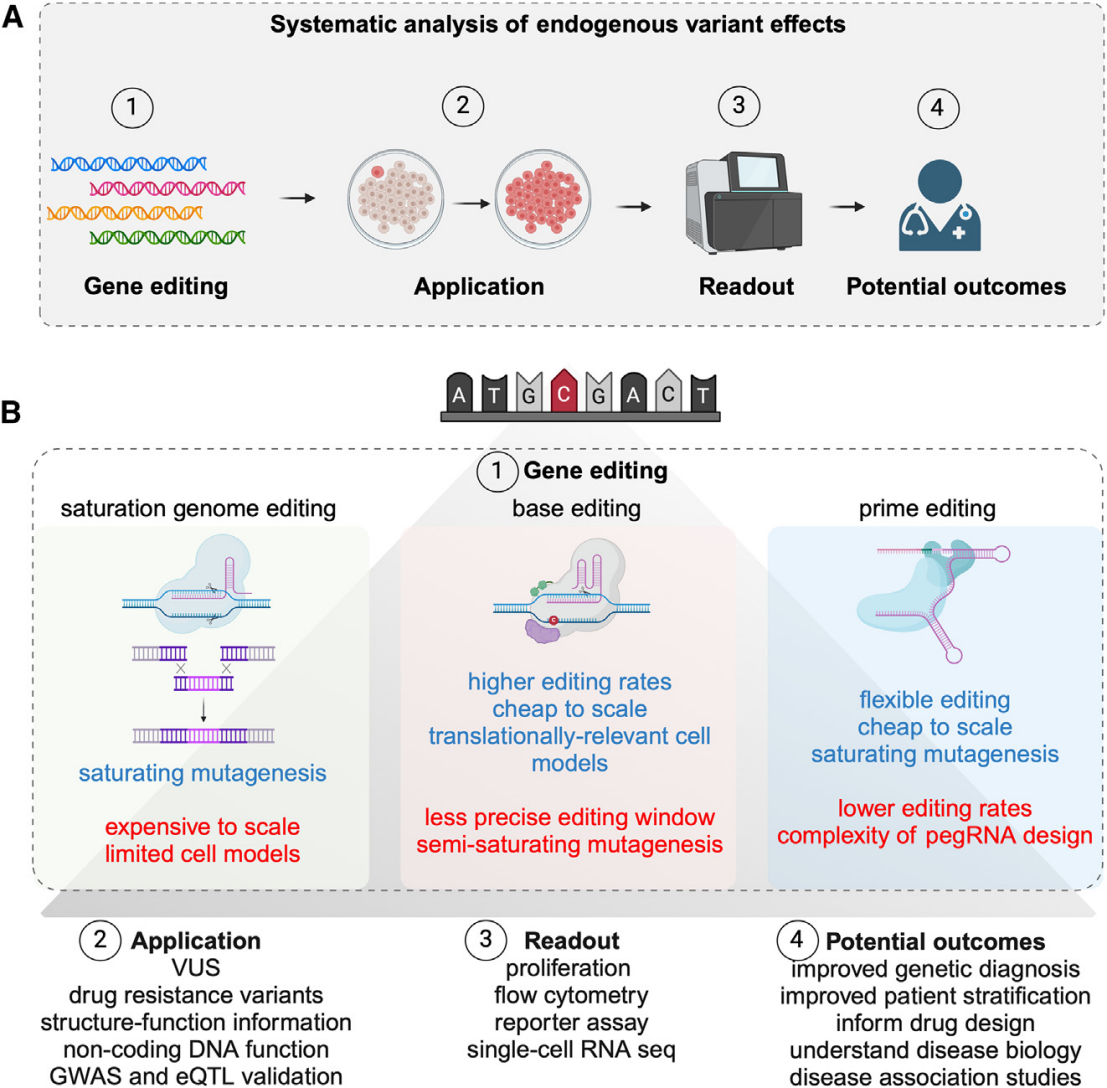
CRISPR-based gene editing methods to analyze endogenous variant function (A) Schematic of a multiplexed assay of variant effect (MAVE) to analyze endogenous variants. These experiments have four main considerations: first, the method of installing the variant using gene editing technologies, which has a bearing on the cell model selected; second, the application, which includes the means of selecting cells based on the phenotype of interest and should be disease relevant; third, the readout for the variant function, which can be proliferation based or include higher-content readouts, such as scRNA-seq; fourth, the outcomes of the variant-function map, which include the potential value of variant interpretation for patients with genetic diseases, including disease association, predicting drug resistance, and patient stratification for appropriate therapies. (B) Outline of the gene editing methods that can be used to install endogenous variants into the genome. The relative advantages and disadvantages of SGE, base editing, and prime editing are highlighted in blue and red, respectively.

Here, we treat endogenous MAVEs as experiments with four key aspects: (1) the gene editing methodology, (2) the application, (3) the phenotypic readout, and (4) the potential translatable outcomes (Figure 1A). We consider the relative merits of state-of-the-art gene editing technologies to study variant function at scale. Furthermore, we discuss how the selected gene editing method can influence subsequent steps in the MAVE, including selecting an appropriate readout and application relevant to the genetic disease in question, to maximize the translation of the resulting data into improved outcomes for patients.

## Saturation genome editing

Saturation genome editing (SGE)^18^ is a technology that uses CRISPR-Cas9 to enable functional analysis of genetic variants at single-nucleotide resolution while maintaining genomic context. For a typical SGE experiment, a guide RNA-Cas9 complex targeted to the genomic region to be edited induces a double-strand DNA break (DSB). The presence of a co-transfected library of variant-harboring repair templates leads to the incorporation of nucleotide changes at the locus through homology-directed repair (HDR). For loci where knockout leads to cell death or a change in fitness, cells with incorporated variants that have an adverse effect on gene function will be less represented within the cultured cell population over time, while variants with a neutral or positive effect are maintained or increased, respectively. Variant count is determined through amplicon sequencing at different time points throughout cell culture, and subsequent informatic analyses convert changes in variant abundance to functional scores, allowing for the study of variant functional effects.

SGE can help guide clinical decision-making and gain fundamental biological insights, in particular, sequence-structure relationships. It also allows scoring of effects that are not only manifested at the protein level. This includes alleles that compromise non-coding and splicing regions, allowing for deleterious effects on endogenous processes such as codon usage, transcription, translation, and post-translational modification, to be included in phenotypic outputs (Figure 1B). In addition, stop-gained and frameshift variants in terminal exons and upstream of non-canonical translational start sites, which might be anticipated to be deleterious, can be assayed for escape of nonsense-mediated mRNA decay (NMD) and alternative start codon usage, respectively.

SGE was first applied in the human near-haploid cell line (HAP1), derived from the KBM-7 chronic myelogenous leukemia cell line.^18^^,^^19^^,^^20^ The HAP1 cell line has been further modified to increase HDR rates by ∼4-fold by limiting the efficiency of the non-homologous DNA end joining (NHEJ) repair pathway.^19^ Haploidy at a locus means that successful editing need only occur at one copy for recessive phenotypes to be manifested, limiting possible confounding effects of the unedited/wild-type (WT) allele. For loss-of-function (LoF) mechanisms, this is a sensible approach, especially for tumor suppressor genes such as *BRCA1*.^19^ However, it has also been observed that HAP1 cells increase in ploidy with culturing, which likely contributes to noise at later time points, even for cells sorted for haploidy pre-screening.^21^ Cell culture optimizations have been shown to lead to a reduction in the diploidization of HAP1 cell cultures;^22^^,^^23^ however, it remains to be seen how much noise unedited alleles introduce in such assays. For genes that are not essential in HAP1 cells, or where cell-type-specific or dominant effects are important, editing of diploid genomes has been applied successfully. This is the case for regions of *CARD11*, assessed in B cells through bi-allelic editing,^24^ where cells were cultured with and without ibrutinib to distinguish gain-of-function (GoF) and LoF variants. Moreover, diploid genomes have also been successfully edited in regions of *NPC1* and *BRCA2*, assayed in HEK293T cells through haploidization at the loci.^25^ In principle, haploidization is appropriate for SGE where cells other than HAP1 are required and efficient biallelic editing is necessary.

When editing in multiplex with SGE, to prevent re-cleavage of incorporated DNA tracts and to increase relative editing efficiency, the inclusion of fixed synonymous/silent variants, termed protospacer adjacent motif (PAM)/protospacer protection edits (PPEs), which are refractory to the Cas9-guide RNA cleavage, are a requirement in all templates. While most fixed synonymous changes would be predicted to have a neutral effect, it is possible that the inclusion of PPEs have confounding effects on distal variant interpretation in some cases. Such effects can be avoided by targeting each locus with two guide RNA-HDR repair library pairs in separate experiments to produce “scarless” SGE maps or mitigated by choosing PPE variants not predicted to alter splicing in the context of distal variants. Furthermore, transient transfection of guide RNAs, rather than transduction, is commonly used in SGE screens.^19^^,^^21^^,^^23^ This approach renders CRISPR machinery ineffective in cultured daughter cells after editing, further reducing repetitive cleavage and the inclusion of unintended compound variants. Last, in downstream NGS analysis and variant calling following SGE, the addition of PPEs in conjunction with the desired library variant can help identify true editing over noise from PCR or sequencing errors in cases where variant allele frequencies are low.

A potential limitation to the more widespread adoption of SGE is the complex nature of the library design. To help address this issue, software packages such as VaLiAnT^26^ have been developed, which allow users to output HDR template sequences containing various mutational consequences over specific genomic ranges. In addition, the analysis and quality control of SGE data, which are generally well modeled as a negative binomial distribution, have been streamlined through the adoption of DESeq2, allowing users to normalize variant changes to appropriate controls, calculate replicate correlations and *p* values, and compute logarithmic fold change (LFC) between variants across multiple time points.^21^^,^^27^

### Essentiality screens with SGE

**Figure 2:**
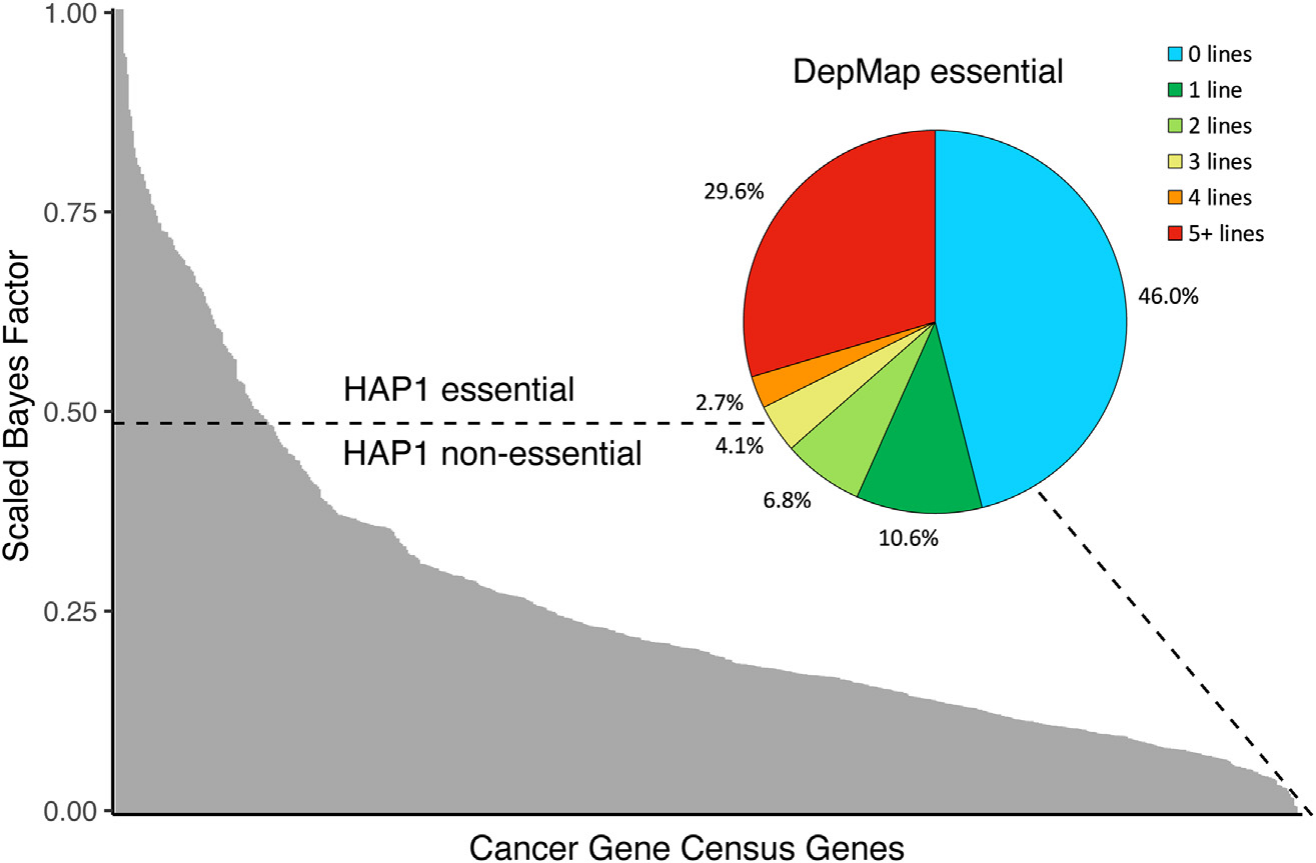
Evaluation of cancer gene essentiality in HAP1 and alternative cell models 85 CGC genes are essential in HAP1 cells. There is a horizontal dashed line at scaled Bayes factor = 0.485, above which genes have a 90% posterior probability of being essential and can be screened with current SGE essential/growth phenotyping.^31^ 355 of 658 genes that are not essential in HAP1 cells are essential in at least one DepMap^30^ cell model, suggesting that essential/growth phenotyping would be possible. 303 of 658 genes would require alternative phenotyping methods downstream of SGE. Data displayed were taken from Hart et al*.*^31^

Mutations play an important role in carcinogenesis; however not all mutations that cause altered function in cancer genes are known, making SGE particularly relevant to oncology. In the absence of functional/supporting data, many variants are classified as VUSs, representing a major challenge to patient management.^17^^,^^28^ A survey of the Cancer Gene Census (CGC) shows that 59% (440 of 743, December 2023) of genes^29^ are predicted to have a strong growth phenotype in HAP1 cells (*n* = 85) or at least one cell line in DepMap (*n* = 355; Figure 2).^30^ 46% of HAP1 non-essential genes (303 of 658), including clinically relevant oncogenes, are not essential in any DepMap model; for these genes, alternative phenotyping will be needed in SGE experiments. Essentiality screens in HAP1 cells are likely to be more phenotypically sensitive than those performed in diploid lines due to the absence of confounding unedited alleles, allowing for LoF mechanisms with a clear correlation to cell death to be measured with high confidence. However, essentiality phenotyping will not be appropriate in all contexts, and there is a need for alternative phenotyping strategies for diverse disease-relevant targets to be developed. Moreover, richer phenotyping approaches, beyond binary classifications, would advance the field and may include coupling genotype to single-cell RNA sequencing (scRNA-seq), as discussed in a later section of this review. However, it is notable that HAP1 essentiality used in SGE screening of *DDX3X*^21^ and distinguishes known cancer and developmental disorder variants as exhibiting compromised function. This indicates that, in these cases at least, essentiality readouts can be informative for different disease phenotypes, even those manifested in organs as complex as the developing fetal brain.

### Future directions for SGE of MAVEs

The expansion of MAVEs has led to the creation of the Impact of Genomic Variation on Function (IGVF), and the Atlas of Variant Effects (AVE) alliance, international consortia that bring together those active in the production of MAVE maps. These consortia help increase collaboration by sharing expertise in the generation of MAVE data and their interpretation and building computational models of variant effect. Associated central repositories of mutagenesis data, such as MAVEdb^14^ and ProteinGym,^32^ facilitate easy access to pre-existing MAVE data. The MAVE registry^33^ also highlights ongoing MAVE projects to effectively coordinate global research efforts. A key aim of AVE is to understand variation in all human genes.^15^ This is a very large undertaking that will face complex challenges in terms of method development, analysis, informatics, and data management. There is much that can be achieved by working openly and collaboratively with a diverse group of organizations and individuals. For example, different approaches to the delivery of SGE reagents into cells have been used across studies. *BRCA1*^19^ and *VHL*^23^ studies have used the delivery of guide RNAs and Cas9-containing plasmids. The SGE study of *DDX3X*^21^ integrated Cas9 into the genome through viral transduction, followed by transient transfection of guide RNAs and HDR templates into polyclonal Cas9-expressing HAP1 cells. The SGE study of *CARD11*^24^ electroporated TMD8 cells with guide RNAs and single-stranded oligodeoxynucleotide repair templates. While experiment-specific requirements will inevitably be necessary, a systematic refinement of SGE protocols will help to standardize a generalizable approach that, together with reagent and analysis optimization, will aid in the adoption and coordination of SGE studies.

SGE data provide a simple and quantitative metric for the discernment of variant effect, which has been demonstrated to be highly sensitive and specific.^19^^,^^21^^,^^23^ In addition, SGE data are also informative in terms of evolutionary function, with regions of high intolerance shown to correlate with high conservation.^21^ The production of multiple variant effect maps for conserved domains in different genes may enable highly accurate predictive models that could reduce the need for further experimentation on a subset of molecular targets. Machine learning models such as EVE (evolutionary model of variant effect)^7^ and AlphaMissense,^34^ which are primarily based on variant evolutionary conservation and protein structural context, could be further refined using data from high-throughput SGE.^7^^,^^34^

The advent of AlphaMissense may raise questions about the relevance of performing SGE at scale; however, empirically derived experimental data are still the gold standard for informing clinical classification. In addition, for both missense and non-coding variants, measuring variant effects in their native cell and genomic contexts provides an advantage over predictive models in terms of physiological relevance, especially in targets with unpredictable sequence-function relationships, such as intrinsically disordered proteins, or for proteins and domains with limited homologs, reducing the power of the multiple sequence alignment component of these models. Moreover, while AlphaMissense has been shown to be highly correlated to MAVE datasets and to demonstrate high sensitivity, it also is less specific than MAVEs (as are other *in silico* predictive tools),^35^ suggesting that experimental functional assays are likely to exhibit fewer false positives—a critical consideration for the clinical application of these data. Importantly, studies have clearly demonstrated the clinical value of SGE data, enabling the resolution of disease kindred pedigrees, aiding patient stratification and clinical management.^36^

## Base editing

Base editing is a CRISPR-Cas9-based gene editing technology that can install programmed SNVs into the genome with minimal introduction of DNA insertions and deletions.^37^ Cytosine base editors (CBEs) can install C>T transition mutations, whereas adenine base editors (ABEs)^38^ can install A>G transition mutations into the genome through guide RNA-mediated recruitment of a nickase Cas9 fused to a cytidine or adenine deaminase, respectively. Therefore, base editing represents an efficient system that does not require exogenous DNA templates or variant libraries, making it an attractive approach for the large-scale, pooled functional screening of variants (Figure 1B).

Base editing has been used successfully to achieve systematic mutagenesis in high-throughput screens to study variant effects in cancer.^39^^,^^40^ One advantage of base editing is that its high efficiency and independence from HDR means that it can be applied in therapeutically relevant cell models, including primary cells, and can therefore be used to investigate the effect of variants in the context of a disease-relevant environmental stimulus, such as therapy. Base editing was recently used to study the effects of variants in the interferon-γ (IFN-γ) pathway on anti-tumor immunity, using guide RNA libraries to tile endogenous genes.^41^ Base editing screens in cancer cell models, including primary tumor organoids, enabled the analysis of how cancer variants affect the sensitivity to IFN-γ and autologous anti-tumor T cells.^41^ Homozygous LoF is required to acquire resistance to IFN-γ signaling,^42^ and base editing is therefore a suitable gene editing strategy for installing these variants to homozygosity in aneuploid cancer cell lines. Moreover, the high efficiency of base editing means that sensitivity is sufficiently high to facilitate drop-out screening (negative selection), allowing for the detection of both LoF and GoF missense mutations across the IFN-γ pathway.^41^ Base editing tiling screens can also be coupled to phenotypes other than proliferation by using fluorescence-activated cell sorting (FACS) to select cells based on the expression of cell surface markers^41^ or fluorescent reporters. For example, the enzymatic activity of variants in the DNA methyltransferase *DNMT3A* were systematically analyzed in a base editing screen using a methylation-sensitive fluorescent reporter.^43^ Base editing has also been used recently to study the effects of DNA variants in 385 genes on effector function in human T cells,^44^ further highlighting the applicability of base editing for endogenous MAVEs in primary cell types.

However, limitations of base editing screens include the lack of editing precision due to the editing activity of the deaminases spanning ∼5 nt within the single-stranded DNA exposed by guide RNA binding (the base editor activity window),^37^ meaning that multiple bases can be edited. Furthermore, the saturation of base editing can be limiting due to the requirement for a PAM in close proximity to the target base and the inability to generate variants other than single-nucleotide transition mutations. These issues have been partially addressed by the development of more precise base editors with engineered deaminase enzymes and by using engineered Cas9 enzymes with more relaxed PAM requirements.^45^^,^^46^ More recently, C>G base editors^47^^,^^48^ and A>C base editors^49^ have been developed to install transversion mutations, expanding the scope of SNVs that can be installed.

## Prime editing

Prime editing is a complementary gene editing tool that uses a reverse transcriptase fused to a nickase Cas9, enabling the direct installation of any sequence less than approximately 50 nt in length.^50^ Furthermore, the saturation of editing achievable is much greater than that of base editing due to the ability to edit sequences farther away from the PAM by modifying the reverse transcription template and primer binding site portions of the prime editing guide RNA (pegRNA). However, scaling up prime editing screens to assess variant effects presents a unique set of challenges (Figure 1B). First, the efficiency of prime editing is generally lower than that of base editing within the activity window,^50^ therefore reducing the signal-to-noise ratio in positive selection screening and reducing the sensitivity to detect deleterious mutations in negative selection screens. Second, the three-component design of pegRNAs means that predicting the efficiency of a given pegRNA is more complicated, making it challenging to select small numbers of highly efficient pegRNAs without extensive empirical testing. However, algorithms for automated pegRNA design are improving and have recently benefitted from machine learning approaches based on large-scale datasets of prime editing outcomes.^51^^,^^52^ Complementary approaches to increase prime editing efficiencies include evolving the prime editor enzyme itself (e.g., PEmax),^53^ engineering the pegRNA to increase its stability,^54^ and disabling mismatch repair machinery to increase the retention of SNV edits in the cell.^53^^,^^55^ Selecting prime-edited cells with FACS by using fluorescent reporters of editing, such as prime editor activity reporter (PEAR),^56^ is an effective orthogonal approach and has also proven to be an effective strategy for selecting cells with high levels of base-editing activity.^57^

Despite these challenges, prime editing has been used successfully at scale to determine variant effects. For example, saturation prime editing was used to classify 978 gene variants in *NPC1*; variants in *NPC1* can cause Niemann-Pick disease type C.^25^ To overcome the limits of prime editing efficiency, targeted haploidization of *NPC1* was performed using Cas9 to eliminate all but one allele, therefore increasing the overall penetrance of prime edits at the *NPC1* locus. To achieve this, the authors exploited the existence of single-nucleotide polymorphisms (SNPs) to achieve single nucleotide-level precision with Cas9. CRISPR guide RNA-assisted reduction of damage (CRISPR GUARD) technology was critical to protect one copy of *NPC1* from being targeted; an approach where a nuclease-dead, truncated guide RNA protects the off-target site from Cas9 cleavage.^58^^,^^59^

More recently, several successful positive selection screens using prime editing in cancer cell models have been reported. For GoF oncogenic and drug resistance mutations, homozygous mutation is not required, making prime editing an attractive approach. For example, genetic resistance to EGFR inhibitors in lung cancer cells was recently investigated by prime editing-mediated installation of 1,220 different variants across seven genes.^60^ Another study analyzed the effect of variants in the commonly mutated tumor suppressor *TP53* using a comprehensive library of over 28,000 pegRNAs.^61^ Interestingly, comparing the effects of endogenous edits to those reported from deep mutational scanning experiments using cDNAs highlighted some key differences from the two methods. These were particularly notable in the oligomerization domain of TP53, suggesting that overexpression of TP53 variants from cDNAs may not reflect the physiological behavior of the native TP53 tetramer and reinforcing the importance of introducing endogenous edits when analyzing variant effects in genes coding for protein complexes.

## Genotyping DNA variants from endogenous MAVEs

We have discussed several advantages of editing the endogenous DNA in MAVEs, but verifying that the correct edits have been installed is important for the interpretation of such screens and can be technically challenging. For example, endogenous variant allele frequency analysis is central to the interpretation of SGE experiments and is also needed to ensure that the intended edits are installed in the context of base editing and prime editing screens. However, for large-scale endogenous MAVEs, it becomes prohibitively expensive to achieve sufficiently high sequencing coverage to measure all variants, especially when editing rates are low (e.g., prime editing) or when there are high rates of unwanted editing by-products, such as insertions or deletions (indels; e.g., SGE). Moreover, scaling amplicon sequencing to multiple loci can be challenging and laborious, ultimately limiting the scale of multi-exon or multi-gene MAVEs.

**Figure 3:**
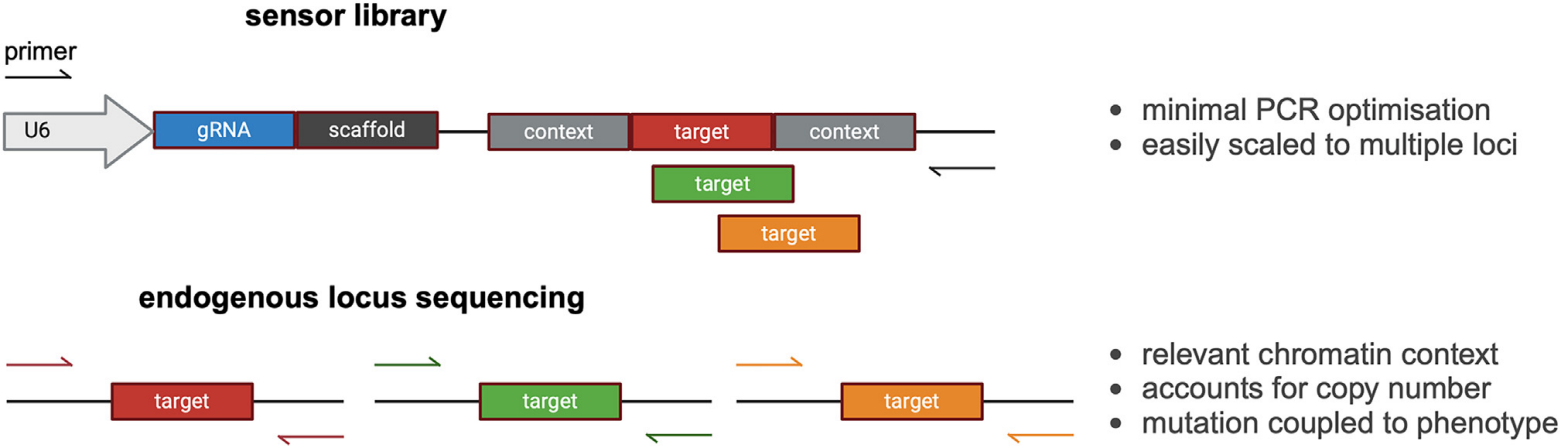
Genotyping strategies for endogenous MAVEs Genotyping of edits in endogenous MAVEs can be performed using sensor libraries or sequencing of the endogenous DNA locus. For sensor libraries, the target sequence is inserted into the guide RNA expression construct, including short regions of flanking DNA context. The construct is incorporated into the genome, and the editing outcomes for all targets are readout as a single, pooled PCR. For endogenous sequencing, multiple primer pairs are required for each locus. Multiplexed PCRs are possible but may require optimization. The advantages of both strategies are highlighted on the right.

To address these issues, sensor systems have been used effectively as a surrogate measure of editing in prime editing^61^ and base editing screens.^62^^,^^63^^,^^64^ The sensor systems rely on incorporating the target sequence into the gene editing delivery vector. Self-targeting gene editing outcomes can then be measured from a single PCR of the vector and read out with NGS^65^^,^^66^ (Figure 3). The sensor systems are useful in detecting and filtering out pegRNAs that do not edit and for monitoring base editing guide RNAs that have unintended bystander edits. However, sensor systems may not be as appropriate for all positive selection screens, where there is the possibility of a disconnect between sensor editing and endogenous editing. For example, rare bystander base edits could have a strong phenotypic effect in the endogenous context leading to enrichment in the screen, but this edit will have no phenotypic effect in the sensor construct and so appear as a minor allele. This effect is less of a concern in prime editing screens due to their high fidelity.^50^^,^^61^^,^^63^ An alternative genotyping method is a multiplexed PCR to assess endogenous edits in a pooled manner (e.g., using the Mission Bio Tapestri platform).^41^^,^^67^ This is an attractive approach, as it can also be applied to genotype single cells and subsequently paired to transcriptomic phenotyping data.^41^^,^^68^

## Phenotypes beyond essentiality

Screening the phenotypic effects of SNVs using readouts such as life-death-based selection or flow cytometry is relatively simple and effective and can be achieved by either directly sequencing the genomic edits introduced in the DNA, as in SGE experiments, or by sequencing the guide RNA as a proxy for editing in base editing or prime editing screens. In many cases, these simple readouts appear to correlate well with data from patients,^19^^,^^21^^,^^23^ but they are not always representative of the disease phenotype and may thus give false positives or negatives. More complex readouts, such as single-cell transcriptomics, are an attractive alternative and, in some cases, have been shown to delineate different functions of the protein, such as the effect of TP53 on cell cycle or apoptosis.^69^ However, it is more challenging to apply SNV screening to more complex readouts, such as transcription of the target gene, single-cell transcriptomics, or imaging-based phenotypic assays. We discuss some of the advances in this area below (Table 1).

**Table 1:** Summary of the different techniques to functionally screen variants using transcriptomic readouts

Technique	Advantages	Disadvantages
Bulk RNA-seq from isogenic lines containing edited SNVs of interest^70^	easy to link genotype to phenotype	time consuming and low throughput, problems with clone-to-clone variation, only bulk RNA-seq data (no information on cell subpopulations)
Arrayed screening for target gene expression (RNA/DNA in SGE experiments, GenIE)^19^^,^^71^	medium throughput and internally controlled so very sensitive to transcription changes	limited to assaying only the transcript in which the variants are located; limited to coding variants, 5′ or 3′ UTR variants; or variants within introns (if nascent RNA is assayed); cannot assay other non-coding variants
Single-cell base editing screens^67^^,^^72^	high-throughput scRNA-seq readout, can assay coding and non-coding variants (only certain edits), can be used in many cell types	requires guide RNA in a suitable location; limited to C>T and A>G mutations; guide RNA identity is used as a proxy for editing events, so poor editing efficiency or bystander editing may create noise or SNV function misassignment
Single-cell prime editing screens; to date, no reported publications	high-throughput scRNA-seq readout, can accurately assay any coding or non-codingvariants	pegRNA identity used as a proxy for editing, so poor efficiency may be a problem
Coupling DNA and RNA in same cells using multiomics approaches^73^^,^^74^^,^^75^	can read out edits in the genomic DNA and obtain scRNA-seq data; some techniques are higher throughput, using split pool or droplet-based approaches	allele dropout rates are often too high to confidently assign SNVs, especially in heterozygotes; large cost of genome sequencing; most are quite low throughput; higher-throughput techniques have even higher allele dropout rates
Reading out edited variants directly from the RNA from single-cell approaches^76^^,^^77^	high-throughput and easy to adapt from current droplet-based workflows using short or long reads	may suffer template switching between SNV and cell barcode during PCR enrichment stage, limited to coding SNVs in highly expressed genes, blind to SNVs causing NMD
Coupling single-cell genotyping and droplet-based scRNA-seq using a cell barcode (scSNV-seq)^68^	high throughput, simple to set up as it uses commercial kits (scGenotyping Mission Bio, scRNA-seq 10× Genomics), can be used for any coding and non-coding SNVs, genotype assignment is good for all alleles	expensive as it requires two commercial kits, requires cell barcoding and cells to divide after editing

## Target gene transcription readouts of endogenous MAVEs

In order to investigate the expression changes of target genes caused by the introduction of SNVs at their endogenous location using CRISPR approaches, several methods have used a comparison of RNA levels of each variant normalized to the level of edits in the DNA to provide a sensitive method for assaying the effects of variants on transcription in a pooled manner. In these cases, the edited SNVs have to be located in the transcribed part of the gene being assayed, allowing the SNV to be detected in both the cDNA and genomic DNA libraries. In a *BRCA1* SGE study,^19^ coding variants were quantified in the RNA (cDNA) and compared to levels of editing in the DNA, and the ratio of abundance was used in order to identify which variants reduce the level of *BRCA1* expression. In a technique called genome engineering-based interrogation of enhancers (GenIE),^71^ the effects of SNVs in non-coding regions or identified from spliceQTLs or fine mapping Alzheimer GWAS variants in the *CLU* gene were screened with a similar cDNA/gDNA comparison. Such techniques are medium throughput and allow many more SNVs to be screened compared to making individual isogenic lines, but the cDNA/gDNA approach is limited to looking at only the transcript in which the SNV is located.

In order to study gene-regulatory elements, high-throughput methods, such as massively parallel reporter assays,^78^ are a powerful way to measure the effects of SNVs on expression of a reporter gene but are limited since they do not replicate the natural genomic and chromatin context. A recent method, GenIE-ATAC, performs editing at endogenous regulatory elements and assays the effect of SNVs on chromatin accessibility^79^ using a ratiometric method analogous to that described above. This method also allows analysis of both changes in target transcript expression and chromatin accessibility upon editing to provide a targeted, multimodal approach.

### Single-cell transcriptomics and high-content readouts for endogenous MAVEs

The methods discussed above can only read out the effect of the SNV on the transcript or regulatory element in which it is located, and it is not possible to obtain the full transcriptome of endogenously edited cells, for which other techniques are required. Overexpression of cDNA variant libraries can provide valuable information to measure the effects of coding SNVs and can be coupled to complex single-cell transcriptomic readouts. For example, by linking the exogenously introduced variant with a barcode that can be read out in single-cell transcriptomics (Perturb-seq), 200 coding variants in *TP53* and *KRAS* have been analyzed by scRNA-seq.^80^ While useful, this approach has limitations since it is not performed with endogenous stoichiometry or genomic context.

### Single-cell base and prime editor screens

Pooled base editor screens coupled to single-cell omics readouts are one way to screen SNVs in their endogenous context. Similar to single-cell CRISPR knockout (KO)/CRISPR interference (Perturb-seq) experiments, such screens detect the guide RNA sequence within each single cell and use it to predict the editing event that has taken place. As well as scale, one major advantage of a single-cell base editor screen is that it allows both coding and non-coding variants to be assayed. As an example, coding variants tiling across the *JAK1* gene were introduced by base editing, coupled to scRNA-seq and resulted in clear transcriptomic differences in components of the IFN-γ signaling pathway.^68^ Morris et al.^72^ used a single-cell base editing screen at the end of their experimental pipeline (base-editing systematic targeting and inhibition of noncoding GWAS loci with single-cell sequencing, bee-STING-seq) to introduce selected non-coding variants from blood trait GWASs and link them to function. By analyzing the full transcriptome, it was possible to assign both genes regulated in *cis* by specific variants as being causative in blood traits as well as the downstream *trans* effector genes and pathways. Another study has performed one of the most comprehensive single-cell base editing screens to date,^67^ in which the authors assayed the effects of non-coding SNVs located in the promoter regions of the *HGB1*/*2* gene and coding SNPs across the *GATA1* gene in primary human hematopoietic stem and progenitor cells (HSPCs).

A move toward prime editing screens linked to a single-cell readout would, in theory, allow the introduction of any SNV at any location, although, at present, these screens are often limited by efficiency of editing. This is a particular problem for single-cell assays in which the guide RNA is a proxy for editing, and, thus, a low editing efficiency means cells assigned to an inefficient guide RNA could still have a WT phenotype even though the SNV is detrimental to gene function. This is also true for single-cell base editing screens, and, additionally, any edits outside of the window of editing can introduce non-predicted SNVs, which could lead to the incorrect assignment of phenotype to SNV. To address these problems, there have been attempts to identify the base edits that have been introduced into the genome using single-cell DNA amplicon sequencing,^67^ but as the authors discuss, this is not linked to a transcriptomic readout in the same cell.

### Pooled single-cell SNV screening

There are a number of methods that have been developed to specifically couple the genotype and phenotype (at the transcriptional level) in the same single cell. They fall into two broad categories: those that amplify from the whole genome and transcriptome from a single cell and those that read out the genotype from the RNA in a single cell. Whole-genome and transcriptome methods such as G&TSeq (genome and transcriptome sequencing),^81^ Simul-seq (simultaneous DNA and RNA sequencing)^82^ and SIDR (simultaneous isolation of genomic DNA and total RNA)^83^ have been used to detect endogenous SNPs, and more recently, improved coverage of the genomic DNA^84^ has allowed CRISPR edits to be detected. However, these methods are plate based, which limits their scalability, with the exception of sci-L3-RNA/DNA (combinatorial indexing and linear amplification co-assay of the genome and transcriptome), which uses split-pool barcoding,^66^ or DEFND-seq (DNA and expression following nucleosome depletion sequencing) which uses a droplet-based approach.^74^ However, all of these approaches suffer not only from the cost of the whole-genome sequencing but also from a relatively high allele dropout rate (<20%–25%), making it difficult to accurately call heterozygous SNPs, which is particularly important after editing. TARGET-seq^75^ addresses this problem by using targeting amplification of DNA and achieves a low allele dropout rate (∼10%), but this is a plate-based approach and, thus, only scalable to relatively small numbers of cells.

The second approach to couple edited genotypes with single-cell transcriptomics has been to read out the variants introduced directly within the RNA. The Genotyping of Transcriptomes (GoT)^76^ method adapts droplet-based single-cell technologies by enriching the region of the transcript that contains the variant by simple PCR or by circularization then PCR, depending on the position of the variant. Long-read sequencing of full-length cDNAs enriched for transcripts of interest (transcript-informed single-cell CRISPR sequencing, TISCC-seq) has also been used to capture variants in different locations along the cDNA,^77^ which the authors have used in conjunction with a single-cell base editor screen. Such techniques are high throughput and so can cover many SNVs of interest, but they are limited to transcribed variants and also to genes with high-enough levels of transcription. Importantly, if SNVs trigger NMD or allele-specific expression, then there will be no transcript to detect, which could mean that these high-effect SNVs are missed or heterozygous alleles misannotated.

Recently, a technique called single-cell SNV sequencing (scSNV-seq) has been developed to combine the robustness of targeted single-cell genotyping with the throughput of droplet-based scRNA-seq.^68^ Here, the authors use a cell barcode that is genotyped alongside the edited region, and the cell barcode can also be read out in the scRNA-seq in a fashion similar to a guide RNA sequence. In this case, the cell barcode links directly to the genotype of that particular cell, enabling precise annotation of the scRNA-seq data. Using this technique, it would be possible to edit both coding and non-coding SNVs and accurately identify heterozygous and homozygous edits, although it does require the cells to be barcoded and to divide after editing has taken place.

These complex transcriptional readouts are not trivial to analyze in terms of assigning genotypes to cells and identifying the true effects of perturbation from the noise inherent to single-cell transcriptomics, especially when the effect is not large. Accordingly, various methodologies are being developed to analyze single-cell CRISPR KO screens,^85^^,^^86^^,^^87^ but additional work will be required to adapt these to SNVs and genes that have more subtle effects on cellular phenotypes.

These advances have allowed the analysis of the complete transcriptome of a cell upon introduction of edited SNVs at endogenous loci, even in primary cells. Further advances in the editing technology, especially improvements to prime editing and its coupling to single-cell omics, have a lot of potential to scale these efforts still further. All of these techniques can, in principle, also be applied to the expanding range of single-cell multiomics readouts, such as full-length transcript sequencing, proteomics, chromatin accessibility, and modifications, that will further expand our understanding of the effects of genetic variation. In the future, these methods could be applied to even more complex and disease-relevant cellular models in a manner to similar KO screens performed in stem-cell-derived cells,^88^ organoids,^89^ and xenografts^90^ and even *in vivo*,^86^ which will provide even more representative readouts for better modeling disease etiology. Another exciting area of future development that will allow even more relevant cellular functions to be assigned to SNVs will be technologies that allow coupling of the effects of editing to imaging-based cellular phenotypes, such as cell painting and high-content imaging.^16^^,^^91^

## Conclusions and future perspectives

The choice of gene editing method has profound implications for the application, readout, and potential translational outcomes of endogenous MAVEs. There are practical trade-offs between disease relevance, the complexity of the experimental system, and the scale and cost of endogenous MAVEs. The investigator has to strike the right balance for each biological question to maximize the generation of informative data for each assay. Fewer compromises will be required with advancements in gene editing technologies, their efficiency in complex model systems (including *in vivo*^92^^,^^93^), and the development of more complex phenotypic readouts, such as single-cell transcriptomics and imaging (Table 1). The most appropriate gene editing approach depends on several factors, such as the most relevant cell model, the nature of the desired edit, the scale and of the experiment, and the expected disease phenotype. The efficiency and precision of the gene editing technologies, as well as the compatibility with downstream readouts, are key considerations (Figure 1A).

Endogenous MAVEs have exciting potential for addressing fundamental and long-standing questions in genomics, such as the role of non-coding regulatory DNA elements.^92^ Equally, the pre-emptive functional assessment of variants associated with disease has exciting potential to aid diagnosis for patients with rare or previously unseen variants and germline variants from single-parent families or where family history is ambiguous. Functional data from MAVEs will be an increasingly important approach to translate the growing wealth of genomics data into informing clinical decision-making in the diagnosis and treatment of genetic diseases.^15^^,^^28^^,^^93^

## References

[1] Halldorsson B.V., Eggertsson H.P., Moore K.H.S., Hauswedell H., Eiriksson O., Ulfarsson M.O., Palsson G., Hardarson M.T., Oddsson A., Jensson B.O. (2022). The sequences of 150,119 genomes in the UK Biobank. Nature.

[2] Chen S., Francioli L.C., Goodrich J.K., Collins R.L., Kanai M., Wang Q., Alföldi J., Watts N.A., Vittal C., Gauthier L.D. (2024). A genomic mutational constraint map using variation in 76,156 human genomes. Nature.

[3] Karczewski K.J., Francioli L.C., Tiao G., Cummings B.B., Alföldi J., Wang Q., Collins R.L., Laricchia K.M., Ganna A., Birnbaum D.P. (2020). The mutational constraint spectrum quantified from variation in 141,456 humans. Nature.

[4] ICGC/TCGA Pan-Cancer Analysis of Whole Genomes Consortium (2020). Pan-cancer analysis of whole genomes. Nature.

[5] Landrum M.J., Kattman B.L. (2018). ClinVar at five years: Delivering on the promise. Hum. Mutat..

[6] Sessa G., Ehlén Å., Von Nicolai C., Carreira A. (2021). Missense Variants of Uncertain Significance: A Powerful Genetic Tool for Function Discovery with Clinical Implications. Cancers.

[7] Frazer J., Notin P., Dias M., Gomez A., Min J.K., Brock K., Gal Y., Marks D.S. (2021). Disease variant prediction with deep generative models of evolutionary data. Nature.

[8] Sollis E., Mosaku A., Abid A., Buniello A., Cerezo M., Gil L., Groza T., Güneş O., Hall P., Hayhurst J. (2023). The NHGRI-EBI GWAS Catalog: knowledgebase and deposition resource. Nucleic Acids Res..

[9] Cook D., Brown D., Alexander R., March R., Morgan P., Satterthwaite G., Pangalos M.N. (2014). Lessons learned from the fate of AstraZeneca’s drug pipeline: a five-dimensional framework. Nat. Rev. Drug Discov..

[10] Gould S.I. (2023). High throughput evaluation of genetic variants with prime editing sensor libraries. bioRxiv.

[11] Lue N.Z., Liau B.B. (2023). Base editor screens for in situ mutational scanning at scale. Mol. Cell.

[12] Gibson T.J., Seiler M., Veitia R.A. (2013). The transience of transient overexpression. Nat. Methods.

[13] Bergendahl L.T., Gerasimavicius L., Miles J., Macdonald L., Wells J.N., Welburn J.P.I., Marsh J.A. (2019). The role of protein complexes in human genetic disease. Protein Sci..

[14] Esposito D., Weile J., Shendure J., Starita L.M., Papenfuss A.T., Roth F.P., Fowler D.M., Rubin A.F. (2019). MaveDB: an open-source platform to distribute and interpret data from multiplexed assays of variant effect. Genome Biol..

[15] Fowler D.M., Adams D.J., Gloyn A.L., Hahn W.C., Marks D.S., Muffley L.A., Neal J.T., Roth F.P., Rubin A.F., Starita L.M., Hurles M.E. (2023). An Atlas of Variant Effects to understand the genome at nucleotide resolution. Genome Biol..

[16] Bock C., Datlinger P., Chardon F., Coelho M.A., Dong M.B., Lawson K.A., Lu T., Maroc L., Norman T.M., Song B. (2022). High-content CRISPR screening. Nat. Rev. Methods Primers.

[17] Starita L.M., Ahituv N., Dunham M.J., Kitzman J.O., Roth F.P., Seelig G., Shendure J., Fowler D.M. (2017). Variant Interpretation: Functional Assays to the Rescue. Am. J. Hum. Genet..

[18] Findlay G.M., Boyle E.A., Hause R.J., Klein J.C., Shendure J. (2014). Saturation editing of genomic regions by multiplex homology-directed repair. Nature.

[19] Findlay G.M., Daza R.M., Martin B., Zhang M.D., Leith A.P., Gasperini M., Janizek J.D., Huang X., Starita L.M., Shendure J. (2018). Accurate classification of BRCA1 variants with saturation genome editing. Nature.

[20] Llargués-Sistac G., Bonjoch L., Castellvi-Bel S. (2023). HAP1, a new revolutionary cell model for gene editing using CRISPR-Cas9. Front. Cell Dev. Biol..

[21] Radford E.J., Tan H.K., Andersson M.H.L., Stephenson J.D., Gardner E.J., Ironfield H., Waters A.J., Gitterman D., Lindsay S., Abascal F. (2023). Saturation genome editing of DDX3X clarifies pathogenicity of germline and somatic variation. Nat. Commun..

[22] Olbrich T., Vega-Sendino M., Murga M., de Carcer G., Malumbres M., Ortega S., Ruiz S., Fernandez-Capetillo O. (2019). A Chemical Screen Identifies Compounds Capable of Selecting for Haploidy in Mammalian Cells. Cell Rep..

[23] Buckley M., Kajba C.M., Forrester N., Terwagne C., Sawyer C., Shepherd S.T., Jonghe J.D., Dace P., Turajlic S., Findlay G.M. (2023). Saturation Genome Editing Resolves the Functional Spectrum of Pathogenic VHL Alleles. bioRxiv.

[24] Meitlis I., Allenspach E.J., Bauman B.M., Phan I.Q., Dabbah G., Schmitt E.G., Camp N.D., Torgerson T.R., Nickerson D.A., Bamshad M.J. (2020). Multiplexed Functional Assessment of Genetic Variants in CARD11. Am. J. Hum. Genet..

[25] Erwood S., Bily T.M.I., Lequyer J., Yan J., Gulati N., Brewer R.A., Zhou L., Pelletier L., Ivakine E.A., Cohn R.D. (2022). Saturation variant interpretation using CRISPR prime editing. Nat. Biotechnol..

[26] Barbon L., Offord V., Radford E.J., Butler A.P., Gerety S.S., Adams D.J., Tan H.K., Waters A.J. (2022). Variant Library Annotation Tool (VaLiAnT): an oligonucleotide library design and annotation tool for saturation genome editing and other deep mutational scanning experiments. Bioinforma. Oxf. Engl..

[27] Love M.I., Huber W., Anders S. (2014). Moderated estimation of fold change and dispersion for RNA-seq data with DESeq2. Genome Biol..

[28] Brnich S.E., Abou Tayoun A.N., Couch F.J., Cutting G.R., Greenblatt M.S., Heinen C.D., Kanavy D.M., Luo X., McNulty S.M., Starita L.M. (2019). Recommendations for application of the functional evidence PS3/BS3 criterion using the ACMG/AMP sequence variant interpretation framework. Genome Med..

[29] Sondka Z., Bamford S., Cole C.G., Ward S.A., Dunham I., Forbes S.A. (2018). The COSMIC Cancer Gene Census: describing genetic dysfunction across all human cancers. Nat. Rev. Cancer.

[30] Behan F.M., Iorio F., Picco G., Gonçalves E., Beaver C.M., Migliardi G., Santos R., Rao Y., Sassi F., Pinnelli M. (2019). Prioritization of cancer therapeutic targets using CRISPR-Cas9 screens. Nature.

[31] Hart T., Tong A.H.Y., Chan K., Van Leeuwen J., Seetharaman A., Aregger M., Chandrashekhar M., Hustedt N., Seth S., Noonan A. (2017). Evaluation and Design of Genome-Wide CRISPR/SpCas9 Knockout Screens. G3 (Bethesda)..

[32] Notin P., Kollasch A.W., Ritter D., van Niekerk L., Paul S., Spinner H., Rollins N., Shaw A., Weitzman R., Frazer J. (2023). ProteinGym: Large-Scale Benchmarks for Protein Design and Fitness Prediction. bioRxiv.

[33] Kuang D., Weile J., Kishore N., Nguyen M., Rubin A.F., Fields S., Fowler D.M., Roth F.P. (2021). MaveRegistry: a collaboration platform for multiplexed assays of variant effect. Bioinforma. Oxf. Engl..

[34] Cheng J., Novati G., Pan J., Bycroft C., Žemgulytė A., Applebaum T., Pritzel A., Wong L.H., Zielinski M., Sargeant T. (2023). Accurate proteome-wide missense variant effect prediction with AlphaMissense. Science.

[35] Ljungdahl A., Kohani S., Page N.F., Wells E.S., Wigdor E.M., Dong S., Sanders S.J. (2023). AlphaMissense Is Better Correlated with Functional Assays of Missense Impact than Earlier Prediction Algorithms. bioRxiv.

[36] Fayer S., Horton C., Dines J.N., Rubin A.F., Richardson M.E., McGoldrick K., Hernandez F., Pesaran T., Karam R., Shirts B.H. (2021). Closing the gap: Systematic integration of multiplexed functional data resolves variants of uncertain significance in BRCA1, TP53, and PTEN. Am. J. Hum. Genet..

[37] Rees H.A., Liu D.R. (2018). Base editing: precision chemistry on the genome and transcriptome of living cells. Nat. Rev. Genet..

[38] Gaudelli N.M., Komor A.C., Rees H.A., Packer M.S., Badran A.H., Bryson D.I., Liu D.R. (2017). Programmable base editing of A⋅T to G⋅C in genomic DNA without DNA cleavage. Nature.

[39] Hanna R.E., Hegde M., Fagre C.R., DeWeirdt P.C., Sangree A.K., Szegletes Z., Griffith A., Feeley M.N., Sanson K.R., Baidi Y. (2021). Massively parallel assessment of human variants with base editor screens. Cell.

[40] Cuella-Martin R., Hayward S.B., Fan X., Chen X., Huang J.W., Taglialatela A., Leuzzi G., Zhao J., Rabadan R., Lu C. (2021). Functional interrogation of DNA damage response variants with base editing screens. Cell.

[41] Coelho M.A., Cooper S., Strauss M.E., Karakoc E., Bhosle S., Gonçalves E., Picco G., Burgold T., Cattaneo C.M., Veninga V. (2023). Base editing screens map mutations affecting interferon-γ signaling in cancer. Cancer Cell.

[42] Shin D.S., Zaretsky J.M., Escuin-Ordinas H., Garcia-Diaz A., Hu-Lieskovan S., Kalbasi A., Grasso C.S., Hugo W., Sandoval S., Torrejon D.Y. (2017). Primary Resistance to PD-1 Blockade Mediated by JAK1/2 Mutations. Cancer Discov..

[43] Lue N.Z., Garcia E.M., Ngan K.C., Lee C., Doench J.G., Liau B.B. (2023). Base editor scanning charts the DNMT3A activity landscape. Nat. Chem. Biol..

[44] Schmidt R., Ward C.C., Dajani R., Armour-Garb Z., Ota M., Allain V., Hernandez R., Layeghi M., Xing G., Goudy L. (2024). Base-editing mutagenesis maps alleles to tune human T cell functions. Nature.

[45] Nishimasu H., Shi X., Ishiguro S., Gao L., Hirano S., Okazaki S., Noda T., Abudayyeh O.O., Gootenberg J.S., Mori H. (2018). Engineered CRISPR-Cas9 nuclease with expanded targeting space. Science.

[46] Walton R.T., Christie K.A., Whittaker M.N., Kleinstiver B.P. (2020). Unconstrained genome targeting with near-PAMless engineered CRISPR-Cas9 variants. Science.

[47] Kurt I.C., Zhou R., Iyer S., Garcia S.P., Miller B.R., Langner L.M., Grünewald J., Joung J.K. (2021). CRISPR C-to-G base editors for inducing targeted DNA transversions in human cells. Nat. Biotechnol..

[48] Koblan L.W., Arbab M., Shen M.W., Hussmann J.A., Anzalone A.V., Doman J.L., Newby G.A., Yang D., Mok B., Replogle J.M. (2021). Efficient C⋅G-to-G⋅C base editors developed using CRISPRi screens, target-library analysis, and machine learning. Nat. Biotechnol..

[49] Chen L., Hong M., Luan C., Gao H., Ru G., Guo X., Zhang D., Zhang S., Li C., Wu J. (2024). Adenine transversion editors enable precise, efficient A⋅T-to-C⋅G base editing in mammalian cells and embryos. Nat. Biotechnol..

[50] Anzalone A.V., Randolph P.B., Davis J.R., Sousa A.A., Koblan L.W., Levy J.M., Chen P.J., Wilson C., Newby G.A., Raguram A., Liu D.R. (2019). Search-and-replace genome editing without double-strand breaks or donor DNA. Nature.

[51] Hsu J.Y., Grünewald J., Szalay R., Shih J., Anzalone A.V., Lam K.C., Shen M.W., Petri K., Liu D.R., Joung J.K., Pinello L. (2021). PrimeDesign software for rapid and simplified design of prime editing guide RNAs. Nat. Commun..

[52] Mathis N., Allam A., Kissling L., Marquart K.F., Schmidheini L., Solari C., Balázs Z., Krauthammer M., Schwank G. (2023). Predicting prime editing efficiency and product purity by deep learning. Nat. Biotechnol..

[53] Chen P.J., Hussmann J.A., Yan J., Knipping F., Ravisankar P., Chen P.F., Chen C., Nelson J.W., Newby G.A., Sahin M. (2021). Enhanced prime editing systems by manipulating cellular determinants of editing outcomes. Cell.

[54] Nelson J.W., Randolph P.B., Shen S.P., Everette K.A., Chen P.J., Anzalone A.V., An M., Newby G.A., Chen J.C., Hsu A., Liu D.R. (2022). Engineered pegRNAs improve prime editing efficiency. Nat. Biotechnol..

[55] Ferreira Da Silva J., Oliveira G.P., Arasa-Verge E.A., Kagiou C., Moretton A., Timelthaler G., Jiricny J., Loizou J.I. (2022). Prime editing efficiency and fidelity are enhanced in the absence of mismatch repair. Nat. Commun..

[56] Simon D.A., Tálas A., Kulcsár P.I., Biczók Z., Krausz S.L., Várady G., Welker E. (2022). PEAR, a flexible fluorescent reporter for the identification and enrichment of successfully prime edited cells. Elife.

[57] Coelho M.A., Li S., Pane L.S., Firth M., Ciotta G., Wrigley J.D., Cuomo M.E., Maresca M., Taylor B.J.M. (2018). BE-FLARE: a fluorescent reporter of base editing activity reveals editing characteristics of APOBEC3A and APOBEC3B. BMC Biol..

[58] Coelho M.A., De Braekeleer E., Firth M., Bista M., Lukasiak S., Cuomo M.E., Taylor B.J.M. (2020). CRISPR GUARD protects off-target sites from Cas9 nuclease activity using short guide RNAs. Nat. Commun..

[59] Rose J.C., Popp N.A., Richardson C.D., Stephany J.J., Mathieu J., Wei C.T., Corn J.E., Maly D.J., Fowler D.M. (2020). Suppression of unwanted CRISPR-Cas9 editing by co-administration of catalytically inactivating truncated guide RNAs. Nat. Commun..

[60] Chardon F.M., Suiter C.C., Daza R.M., Smith N.T., Parrish P., McDiarmid T., Lalanne J.B., Martin B., Calderon D., Ellison A. (2023). A Multiplex, Prime Editing Framework for Identifying Drug Resistance Variants at Scale. bioRxiv.

[61] Gould S.I., Wuest A.N., Dong K., Johnson G.A., Hsu A., Narendra V.K., Atwa O., Levine S.S., Liu D.R., Sánchez Rivera F.J. (2022). High Throughput Evaluation of Genetic Variants with Prime Editing Sensor Libraries. bioRxiv.

[62] Kim Y., Lee S., Cho S., Park J., Chae D., Park T., Minna J.D., Kim H.H. (2022). High-throughput functional evaluation of human cancer-associated mutations using base editors. Nat. Biotechnol..

[63] Sánchez-Rivera F.J., Diaz B.J., Kastenhuber E.R., Schmidt H., Katti A., Kennedy M., Tem V., Ho Y.J., Leibold J., Paffenholz S.V. (2022). Base editing sensor libraries for high-throughput engineering and functional analysis of cancer-associated single nucleotide variants. Nat. Biotechnol..

[64] Pallaseni A., Peets E.M., Koeppel J., Weller J., Vanderstichele T., Ho U.L., Crepaldi L., van Leeuwen J., Allen F., Parts L. (2022). Predicting base editing outcomes using position-specific sequence determinants. Nucleic Acids Res..

[65] Shen M.W., Arbab M., Hsu J.Y., Worstell D., Culbertson S.J., Krabbe O., Cassa C.A., Liu D.R., Gifford D.K., Sherwood R.I. (2018). Predictable and precise template-free CRISPR editing of pathogenic variants. Nature.

[66] Allen F., Crepaldi A., Alsinet C., Strong A.J., Kleshchevnikov V., De Angeli P., Páleníková P., Khodak A., Kiselev V., Kosicki M., Bassett A.R. (2019). Predicting the mutations generated by repair of Cas9-induced double-strand breaks. Nat. Biotechnol..

[67] Martin-Rufino J.D., Castano N., Pang M., Grody E.I., Joubran S., Caulier A., Wahlster L., Li T., Qiu X., Riera-Escandell A.M. (2023). Massively parallel base editing to map variant effects in human hematopoiesis. Cell.

[68] Cooper S.E., Coelho M.A., Strauss M.E., Gontarczyk A.M., Wu Q., Garnett M.J., Marioni J.C., Bassett A.R. (2023). High-Throughput Phenotyping of Single Nucleotide Variants by Linking Transcriptomes to Genotypes in Single Cells. bioRxiv.

[69] Xu H. (2023). Single Cell Sequencing as a General Variant Interpretation Assay. bioRxiv.

[70] Bello E., Long K., Iwama S., Steer J., Cooper S., Alasoo K., Kumasaka N., Schwartzentruber J., Panousis N.I., Bassett A. (2023). An Alzheimer’s Disease-Associated Common Regulatory Variant in PTK2B Has Causal Effects on Microglial Function. bioRxiv.

[71] Cooper S.E., Schwartzentruber J., Bello E., Coomber E.L., Bassett A.R. (2020). Screening for functional transcriptional and splicing regulatory variants with GenIE. Nucleic Acids Res..

[72] Morris J.A., Caragine C., Daniloski Z., Domingo J., Barry T., Lu L., Davis K., Ziosi M., Glinos D.A., Hao S. (2023). Discovery of target genes and pathways at GWAS loci by pooled single-cell CRISPR screens. Science.

[73] Yin Y., Jiang Y., Lam K.W.G., Berletch J.B., Disteche C.M., Noble W.S., Steemers F.J., Camerini-Otero R.D., Adey A.C., Shendure J. (2019). High-Throughput Single-Cell Sequencing with Linear Amplification. Mol. Cell.

[74] Olsen T.R., Talla P., Furnari J., Bruce J.N., Canoll P., Zha S., Sims P.A. (2023). Scalable co-sequencing of RNA and DNA from individual nuclei. bioRxiv.

[75] Rodriguez-Meira A., Buck G., Clark S.A., Povinelli B.J., Alcolea V., Louka E., McGowan S., Hamblin A., Sousos N., Barkas N. (2019). Unravelling Intratumoral Heterogeneity through High-Sensitivity Single-Cell Mutational Analysis and Parallel RNA Sequencing. Mol. Cell.

[76] Nam A.S., Kim K.T., Chaligne R., Izzo F., Ang C., Taylor J., Myers R.M., Abu-Zeinah G., Brand R., Omans N.D. (2019). Somatic mutations and cell identity linked by Genotyping of Transcriptomes. Nature.

[77] Kim H.S., Grimes S.M., Chen T., Sathe A., Lau B.T., Hwang G.H., Bae S., Ji H.P. Direct measurement of engineered cancer mutations and their transcriptional phenotypes in single cells. Nat. Biotechnol. 2023;123. doi:10.1038/s41587-023-01949-8. Online ahead of print. 10.1038/s41587-023-01949-8 PMC11324510 37697151

[78] Inoue F., Kircher M., Martin B., Cooper G.M., Witten D.M., McManus M.T., Ahituv N., Shendure J. (2017). A systematic comparison reveals substantial differences in chromosomal versus episomal encoding of enhancer activity. Genome Res..

[79] Cooper S., Schwartzentruber J., Coomber E.L., Wu Q., Bassett A. (2023). Screening for functional regulatory variants in open chromatin using GenIE-ATAC. Nucleic Acids Res..

[80] Ursu O., Neal J.T., Shea E., Thakore P.I., Jerby-Arnon L., Nguyen L., Dionne D., Diaz C., Bauman J., Mosaad M.M. (2022). Massively parallel phenotyping of coding variants in cancer with Perturb-seq. Nat. Biotechnol..

[81] Macaulay I.C., Haerty W., Kumar P., Li Y.I., Hu T.X., Teng M.J., Goolam M., Saurat N., Coupland P., Shirley L.M. (2015). G&T-seq: parallel sequencing of single-cell genomes and transcriptomes. Nat. Methods.

[82] Reuter J.A., Spacek D.V., Pai R.K., Snyder M.P. (2016). Simul-seq: combined DNA and RNA sequencing for whole-genome and transcriptome profiling. Nat. Methods.

[83] Han K.Y., Kim K.T., Joung J.G., Son D.S., Kim Y.J., Jo A., Jeon H.J., Moon H.S., Yoo C.E., Chung W. (2018). SIDR: simultaneous isolation and parallel sequencing of genomic DNA and total RNA from single cells. Genome Res..

[84] Gonzalez-Pena V., Natarajan S., Xia Y., Klein D., Carter R., Pang Y., Shaner B., Annu K., Putnam D., Chen W. (2021). Accurate genomic variant detection in single cells with primary template-directed amplification. Proc. Natl. Acad. Sci. USA.

[85] Yang L., Zhu Y., Yu H., Cheng X., Chen S., Chu Y., Huang H., Zhang J., Li W. (2020). scMAGeCK links genotypes with multiple phenotypes in single-cell CRISPR screens. Genome Biol..

[86] Jiang L., Dalgarno C., Papalexi E., Mascio I., Wessels H.H., Yun H., Iremadze N., Lithwick-Yanai G., Lipson D., Satija R. (2024). Systematic Reconstruction of Molecular Pathway Signatures Using Scalable Single-Cell Perturbation Screens. bioRxiv.

[87] Replogle J.M., Saunders R.A., Pogson A.N., Hussmann J.A., Lenail A., Guna A., Mascibroda L., Wagner E.J., Adelman K., Lithwick-Yanai G. (2022). Mapping information-rich genotype-phenotype landscapes with genome-scale Perturb-seq. Cell.

[88] Tian R., Abarientos A., Hong J., Hashemi S.H., Yan R., Dräger N., Leng K., Nalls M.A., Singleton A.B., Xu K. (2021). Genome-wide CRISPRi/a screens in human neurons link lysosomal failure to ferroptosis. Nat. Neurosci..

[89] Liang J., Wei J., Cao J., Qian J., Gao R., Li X., Wang D., Gu Y., Dong L., Yu J. (2023). In-organoid single-cell CRISPR screening reveals determinants of hepatocyte differentiation and maturation. Genome Biol..

[90] Liu S.J., Pak J., Zou C., Casey-Clyde T., Borah A.A., Wu D., Seo K., O’Loughlin T., Lim D.A., Ozawa T., Berger M.S. (2023). In Vivo Perturb-Seq of Cancer and Immune Cells Dissects Oncologic Drivers and Therapy Response. bioRxiv.

[91] Feldman D., Singh A., Schmid-Burgk J.L., Carlson R.J., Mezger A., Garrity A.J., Zhang F., Blainey P.C. (2019). Optical Pooled Screens in Human Cells. Cell.

[92] Zhang X., Meyerson M. (2020). Illuminating the noncoding genome in cancer. Nat. Cancer.

[93] Tavtigian S.V., Greenblatt M.S., Harrison S.M., Nussbaum R.L., Prabhu S.A., Boucher K.M., Biesecker L.G., ClinGen Sequence Variant Interpretation Working Group ClinGen SVI (2018). Modeling the ACMG/AMP variant classification guidelines as a Bayesian classification framework. Genet. Med..

